# Overcoming Immune Exclusion in a Murine Model of Cirrhotic Hepatocellular Carcinoma via Cryoablation: Insights from Molecular Extracellular pH Imaging

**DOI:** 10.21203/rs.3.rs-7879236/v1

**Published:** 2025-12-03

**Authors:** Daniel Coman, Jessica Gois Santana, Annabella Shewarega, David Nam, Marie Louise Löffler, Tabea Kao, Xuchen Zhang, Vinzent Kahl, Fahmeed Hyder, Julius Chapiro

**Affiliations:** Yale University

## Abstract

Solid tumors, including hepatocellular carcinoma (HCC), arises most often in cirrhotic livers, where immune exclusion and metabolic reprogramming drive extracellular acidosis (the “Warburg effect”) and create an immunosuppressive tumor microenvironment (TME). This study applied a non-invasive MR Spectroscopic Imaging method called Biosensor Imaging of Redundant Deviation in Shifts (BIRDS) to quantify extracellular pH (pH_e_) dynamics in a mouse model of cirrhosis-associated HCC. Forty-two Mdr2^−^/^−^ mice received chronic carbon tetrachloride (CCl_4_), inducing cirrhosis and HCC, confirmed by contrast-enhanced MR and histology. BIRDS revealed significantly lower tumor pH_e_ in untreated tumors (6.78 ± 0.3) compared with liver parenchyma (7.17 ± 0.02). Cryoablation induced tumor pH_e_ normalization (7.08 ± 0.03), coinciding with downregulation of metabolic markers and increased T-cell and macrophage infiltration. These results demonstrate that BIRDS enables non-invasive monitoring of the metabolic and immunologic response to cryoablation in HCC within cirrhotic livers. Cryoablation-induced re-normalization of tumor acidity, coupled with enhanced immune activity, suggests a favorable therapeutic outcome and establishes pH_e_ imaging as a tool for assessing treatment efficacy in acidic TMEs.

## Introduction

A longstanding unanswered question in cancer therapy revolves around understanding what factors contribute to progression of certain cancers while others remain dormant or regress. Hepatocellular carcinoma (HCC) stands as a prime example, ranking as the third most common cause of cancer-related deaths globally^[1]^. Its incidence rates are on the rise in both the United States and Europe, particularly among individuals with underlying chronic liver disease^[2]^. HCC prognosis hinges on both the tumor stage and the quality and function of the underlying liver^[3]^. Unfortunately, 60% of patients are diagnosed in the intermediate to advanced stages, for which curative treatment options are scarce^[4–6]^. Following the success of IMbrave 150 trial, the most recent Barcelona Clinic Liver Cancer staging guidelines have incorporated immune checkpoint inhibitors (ICIs) as the first-line treatment for advanced-stage HCC^[7]^. However, although successful in other tumors, clinical trials using ICIs (CheckMate 040 and 459) have achieved sustained responses in only a minority of patients^[8, 9]^. The primary cause of decreased efficacy in immunotherapies for HCC is the profoundly immunosuppressive tumor microenvironment’s (TME), which is shaped by chronic hepatic inflammation, the tumor’s pronounced hypermetabolism, and cellular adaptations that suppress local immune responses^[9]^.

A central feature of metabolic reprogramming is aerobic glycolysis, first described by Otto Warburg^[11]^. His discovery laid the foundation for the field of tumor metabolism, revealing that excessive extracellular acidosis in the TME, caused by aerobic glycolysis (the “Warburg effect”), contributes to T-cell exhaustion and impaired immune function^[12, 13]^. The resulting acidic extracellular pH (pH_e_) within the TME also promotes a tumor-supportive phenotype in macrophages, reinforcing an immunosuppressive TME^[14, 15]^. Locoregional therapies, such as thermal ablative techniques, have demonstrated the ability to activate the immune system^[16–18]^. In rare instances, cancer patients have experienced immune-mediated regression of untreated metastases following the local application of cryoablation^[19]^. Cryotherapy consists of freeze-thaw cycles where the tumor tissue becomes necrotic after ablation with a temperature of at least − 40°C^[20]^. The freeze-thaw cycles create a more pronounced inflammatory response than other ablation techniques (e.g., radiofrequency ablation) as they preserve the TME’s structure, allowing a better exposure of tumor antigens to the immune system and thus facilitating a more robust anti-tumor response^[19, 21]^. However, in most cases, the induced immune response is insufficient to completely destroy the remaining viable cancer cells, particularly at sites distant from the ablation core^[17]^. Considering the rapidly evolving landscape of cancer immunotherapy for HCC, combining treatment modalities has been identified as a key priority to enhance the therapeutic effects of cryoablation^[22, 23]^.

Current strategies that combine locoregional and systemic therapies in HCC are not yet guided by biomarkers or tailored to the TME characteristics^[24]^. Given the complexity of the TME and the prominent role of hypermetabolism in driving immune suppression, there is a critical need for non-invasive imaging tools to monitor metabolic features that influence therapeutic outcomes. In this context, cryoablation extends beyond local tumor destruction technique but also holds potential as an immunological sensitizer. By inducing localized necrosis and releasing tumor antigens, cryoablation can act as an “immune vaccine,” particularly in advanced-stage disease, where immune exclusion remains a dominant barrier^[19, 21]^. Rather than serving as definitive therapy, cryoablation may therefore prime the TME for improved responsiveness to immunotherapies. Thus, this study proposes using Biosensor Imaging of Redundant Deviation in Shifts (BIRDS)^[25–29]^ to non-invasively monitor extracellular pH (pH_e_) as a surrogate of cryoablation-induced metabolic and immune changes in a cirrhotic mouse model of HCC. BIRDS with TmDOTP^5−^ has been validated for pH_e_ imaging in murine brain tumors on preclinical scanners^[25–31]^ and in rabbit liver tumors on clinical scanners^[32, 33]^. Building on these advances, the present work evaluates whether cryoablation-induced shifts in pH_e_ can serve as a biomarker of enhanced immunomodulatory activity.

## Results

### CCl_4_ treatment induces liver cirrhosis and early HCC onset in MD2 knockout mice

The development and progression of HCC were assessed in both spontaneous and chemically induced models. As shown in Fig. 2A, spontaneous liver tumors developed in aged mice (~ 18 months) without any chemical induction. Gross examination revealed a distinct nodule on the anterior lobe, and histological analysis confirmed a neoplastic lesion. IHC staining for CD3, CD68, and CD206 identified immune cell infiltration, primarily for the M2 polarized macrophages, suggesting a pro-tumorigenic microenvironment in spontaneous arising tumors. In contrast, Fig. 2B depicts early HCC development in a chemically induced model. All treated mice developed visible focal lesions in a well-established cirrhotic liver after 24 weeks of CCl_4_ treatment. The treatment regimen followed an administration protocol described by Kim et al.^[35]^, in which escalating doses of CCl_4_ (50% (vol/vol) in olive oil) were administered three times per week for 20 weeks (see Appendix E1 for additional details). Mice were monitored and sacrificed at predefined time points: at the appearance of the first lesions (week 24), and at 4, 8, and 12 weeks following CCl_4_ withdrawal, to examine tumor progression and the evolution of fibrosis in the cirrhotic background. By week 24, livers exhibited a cirrhotic background with early neoplastic nodules. Histological evaluation with H&E, Masson’s trichome, and reticulin staining confirmed the presence of cirrhosis, while CD34 IHC indicated neovascularization associated with early malignant transformation. Progressive tumor burden and architectural distortion of the liver over 1-, 2-, and 3-month recovery intervals (Fig. 2C) suggest persistent and advancing HCC in the context of liver cirrhosis. Together, these results illustrate that while spontaneous HCC develops late in untreated Mdr2^−^/^−^ mice, chronic CCl_4_ accelerates tumor initiation and progression within 6 months. The resulting model recapitulates both inflammatory and fibrotic cues central to human cirrhotic HCC and enables the longitudinal tracking of disease progression following injury cessation.

### Longitudinal monitoring reveals normalization of tumor in response to cryoablation

To assess the impact of cryoablation on the pH_e_ of TME in HCC, BIRDS with MRSI using TmDOTP^5−^ was performed to obtain pH_e_ maps in control and cryoablated mice livers. Representative anatomical T_1_-weighted images and corresponding pH_e_ maps of untreated and cryoablated tumors are shown in Fig. 3A. Given the multifocal nature of HCC in the Mdr2−/− model, cryoablation was applied to a single visible lesion, while additional tumors remained untreated and served as internal controls. Untreated HCC-bearing mice demonstrated lower tumor pH_e_ compared to liver parenchyma (pH_e_ = 6.78 ± 0.3 vs 7.17 ± 0.02; p < 0.0001). cCryo of the targeted lesion increased tumor pH_e_ towards normalization (pH_e_ = 7.08 ± 0.03) compared to untreated (p < 0.0001). The pH_e_ of tumor edge (7.13 ± 0.08) was slightly higher than whole tumor pH_e_ (7.05 ± 0.06; p < 0.001). Quantitative analysis of pooled (n = 6 in each group) tumor pH_e_ values further revealed a significant difference between groups (Fig. 3B). Cryoablated tumors (pH_e_ = 7.09 ± 0.01) displayed a clear shift towards a more alkaline TME compared to untreated tumors (pH_e_ = 6.79 ± 0.37; p = < 0.0001). In contrast, pHe values in non-tumor liver tissue remained comparable between groups (7.16 ± 0.01 vs 7.18 ± 0.08; p = 0.41), indicating that the observed pH_e_ modulation was specific to the tumor compartment and not attributable to systemic or liver-wide changes.

### Cryoablation downregulates tumor metabolic markers and overcomes immunological exclusion

Cryoablation-driven immune and metabolic changes in HCC were assessed through IHC analysis. The lysosomal marker LAMP-2 and glycolytic enzyme hexokinase-2 (HK2) were both robustly elevated in untreated tumors, whereas cryoablated tumors exhibited a marked reduction in staining intensity for both markers (Fig. 4A). Cryoablation area of necrosis was confirmed on the H&E staining. Quantitative image analysis confirmed a significant reduction in the tumor-to-liver ratio of LAMP-2 staining in treated mice compared to untreated (mean ± SD: 1.53 ± 0.27 vs 6.285 ± 0.21; p = < 0.0001) (Fig. 4B). Similarly, HK2 positivity was significantly reduced in treated tumors compared to untreated (1.56 ± 0.25 vs 5.24 ± 0.68; p = < 0.01), suggesting lower glycolytic activity, likely attributed to the loss of viable tumor cells within the cryoablation zone as evidenced by histological necrosis.

### Incomplete cryoablation promotes immune effector recruitment while reduces immunosuppressive phenotypes

Incomplete cryoablation (iCryo) was employed to preserve peripheral tumor viability while ablating the core to investigate cryoablation-induced immune cell recruitment and phenotypic shift in non-necrotic tumor regions (Fig. 5). T_1_-weighted anatomical MR imaging confirmed tumor presence in both untreated and treated groups, with partial tumor ablation observed in the iCryo cohort. The corresponding pH_e_ maps revealed regions of persistent acidity alongside areas of partial pH neutralization within the treated tumors. Histological analysis with H&E staining showed a necrotic tumor core surrounded by a viable, densely cellular tumor rim in the iCryo group, delineating the transition between ablated and non-ablated tumor zones. Histopathologic analysis revealed significant changes in immune cell marker expression between untreated and iCryo-treated tumors. CD68^+^ macrophage infiltration was markedly increased in both Cryo and iCryo groups relative to controls, with the iCryo group showing a tumor-to-liver ratio of 6.00 ± 4.37 vs. 1.63 ± 0.84 in controls (p = 0.0004) (Fig. 5B). CD8^+^ cytotoxic T cell infiltration was also enhanced in both cryoablation groups (Cryo: 4.79 ± 1.60; iCryo: 5.17 ± 0.70) compared to untreated tumors (1.48 ± 0.52; p < 0.0001), with T cell infiltration slightly more elevated in the iCryo groups. Conversely, the expression of CD206, a marker associated with immunosuppressive M2 macrophages, was significantly reduced in the treated tumors (iCryo: 2.53 ± 1.02; control: 5.13 ± 0.89; p = 0.0009), indicating a shift away from an immunosuppressive macrophage phenotype. These findings demonstrate that cryoablation induces spatially localized changes in tumor pH_e_ and promotes infiltration of immune effector cells while reducing markers of immune suppression, collectively reshaping the TME following treatment.

## Discussion

This study demonstrates that tumor cryoablation induces significant remodeling of the HCC TME, characterized by normalization of tumor pH_e_, suppression of metabolic activity, and reversal of immune exclusion. Leveraging the molecular imaging BIRDS platform for high-resolution, voxel-wise pH_e_ mapping, cryoablation was shown to effectively neutralize tumor acidosis. These physicochemical changes were accompanied by enhanced infiltration of immune effector cells and reduced expression of glycolytic and lysosomal metabolic markers within the TME. This is the first preclinical investigation to demonstrate cryoablation-induced immunometabolic modulation in an oncogenic HCC model with underlying liver cirrhosis. The use of Mdr2^−^/^−^ mice combined with CCl_4_ exposure recapitulates the chronic hepatic inflammation, fibrosis, and immune dysfunction that typifies the TME of human HCC^[36]^. Unlike commonly used subcutaneous or orthotopic syngeneic models that lack cirrhotic architecture and immune complexity, this model provides a clinically relevant platform for probing therapeutic mechanisms in the context of chronic liver disease.

Untreated HCC tumors exhibited a highly acidic extracellular environment relative to adjacent liver parenchyma, consistent with a glycolytically driven metabolic phenotype and the Warburg effect^[37, 38]^. Following cryoablation, pH_e_ values within the tumor shifted toward physiological neutrality, accompanied by histopathologic evidence of decreased expression of hexokinase-2 (HK2) and lysosome-associated membrane protein 2 (LAMP-2), indicating downregulation of glycolytic flux and lysosomal activity. These findings are consistent with previous results describing the metabolic vulnerability of HCC and underscore the capability of cryoablation to disrupt oncogenic metabolic circuits critical for immune evasion and tumor survival^[39]^.

In the setting of iCryo, which spares peripheral tumor zones from full necrosis, increased infiltration of CD8^+^ cytotoxic T lymphocytes and CD68^+^ macrophages were observed, alongside a reduction in CD206^+^ M2-like macrophages. These changes suggest a phenotypic shift away from an immunosuppressive to a pro-inflammatory TME following ablative intervention. Such findings are consistent with reports describing cryoablation as a modulator of local immunity through enhanced antigen release and preservation of tissue architecture^[40]^. Notably, pH_e_ measurements revealed persistent acidic pockets in the tumor periphery, despite central neutralization. This heterogeneity may reflect variable thermal penetration, stromal insulation, or residual tumor metabolism, underscoring the need for precision-guided intervention strategies. Prior preclinical studies have suggested that partial cryoablation may foster an immunogenic niche conducive to T-cell priming with or without pre-existing tumor immunogenicity^[41]^.

Notwithstanding, the immune influx observed after iCryo in the cirrhotic Mdr2^−^/^−^ model contrasts with findings from non-cirrhotic models, where matrix modulation was required to achieve similar effects^[42]^. This discrepancy may be explained by the distinct immune microenvironment of cirrhotic livers. Chronic liver injury and fibrosis remodel the stromal and vascular architecture, establishing a pro-inflammatory niche that both restricts effector activity and sustains chronic immune activation^[43, 44]^. Notably, recent studies have also shown that fibrotic immune remodeling can generate a T cell–inflamed microenvironment that enhances responsiveness to perturbations, including immunotherapy and local interventions^[40]^. Thus, the cirrhotic stroma may substitute for exogenous ECM modulation in facilitating immune infiltration after cryoablation, highlighting the importance of underlying liver disease in shaping therapeutic responses^[45, 46]^.

The introduction of extracellular pH_e_ as a functional, imaging-based biomarker for TME characterization offers a novel approach for treatment monitoring. Its responsiveness to both metabolic and immunologic shifts makes it a promising candidate for guiding combination therapies, particularly in patients with cirrhosis where therapeutic indices are narrow and systemic toxicity must be minimized. Real-time pH_e_ monitoring may ultimately enable personalized timing of systemic therapies, including immune checkpoint inhibitors, in conjunction with ablative modalities^[47]^. Additionally, the integration of cryoablation with voxel-level pH_e_ imaging represents a technological advancement in cancer theranostics. By identifying extracellular pH_e_ as a sensitive indicator of metabolic suppression and immune activation, this study establishes a foundation for future biomarker-driven clinical protocols. The data support the potential for combining cryoablation with systemic immunotherapies in a biomarker-guided manner, enhancing the therapeutic efficacy and selectivity in patients with HCC and underlying cirrhosis. Given the translational relevance of the imaging methodology and disease model, these findings have direct implications for the design of next-generation clinical trials.

Although this study shows the potential of pH_e_ imaging with BIRDS, some limitations should be addressed. While voxel-wise data provides unprecedented insights into metabolic heterogeneity, co-localization with immune cell distribution remains indirect. Temporal profiling of immune responses was limited to early post-treatment windows, and future studies should assess the durability of immune activation and the potential for systemic anti-tumor immunity, such as cytokine profiling. In the future, comparative analysis with other ablative modalities such as radiofrequency or microwave ablation should be performed to further delineate the unique immunometabolic impact of cryo-based therapies.

In summary, this study establishes extracellular pH imaging using the BIRDS platform as a powerful, non-invasive tool to quantify the metabolic and immune consequences of cryoablation in cirrhotic hepatocellular carcinoma. The identification of extracellular pH_e_ as a dynamic, non-invasive biomarker provides a mechanistic and translational framework for optimizing the integration of locoregional and systemic therapies. These findings support the advancement of pH_e_-guided, combinatorial treatment paradigms that address both metabolic and immunologic barriers to effective HCC therapy.

## Methods

### HCC animal model with cirrhotic background

**Figure 1:**
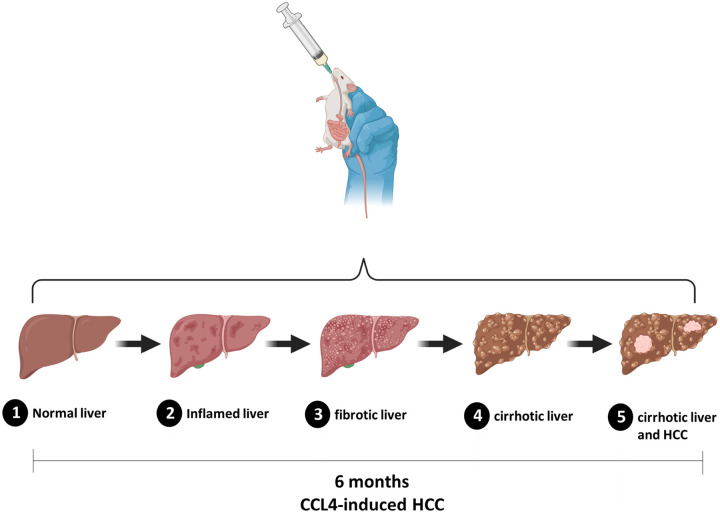
Schematic showing progression of CCl_4_-induced hepatocarcinogenesis in Mdr2^−^/^−^ mice. Mdr2 knockout mice subjected to chronic carbon tetrachloride (CCl_4_) exposure develop progressive liver disease over 6 months, transitioning from normal liver to inflammation, fibrosis, cirrhosis, and ultimately hepatocellular carcinoma (HCC) within a cirrhotic microenvironment. This model reflects the human pathophysiological continuum of liver carcinogenesis and enables evaluation of therapeutic interventions in an immune-competent, fibrotic liver setting.

All experimental protocols were approved by the Institutional Animal Care and Use Committee at [blinded]. All methods were performed in accordance with the ARRIVE guidelines and in accordance with the relevant guidelines and regulations. Multidrug resistance gene 2 (Mdr2^−^/^−^) mice were kindly provided by X X ([blinded]). At 3 weeks of age, mice received repetitive oral administration of escalating doses of carbon tetrachloride (CCl_4_), 50% (vol/vol) in olive oil, three times per week for 24 weeks for CCl_4_-induced early onset of tumor growth on a cirrhotic liver background (Fig. 1) (see Appendix E1 for additional details).

### Experimental design

**Figure 2:**
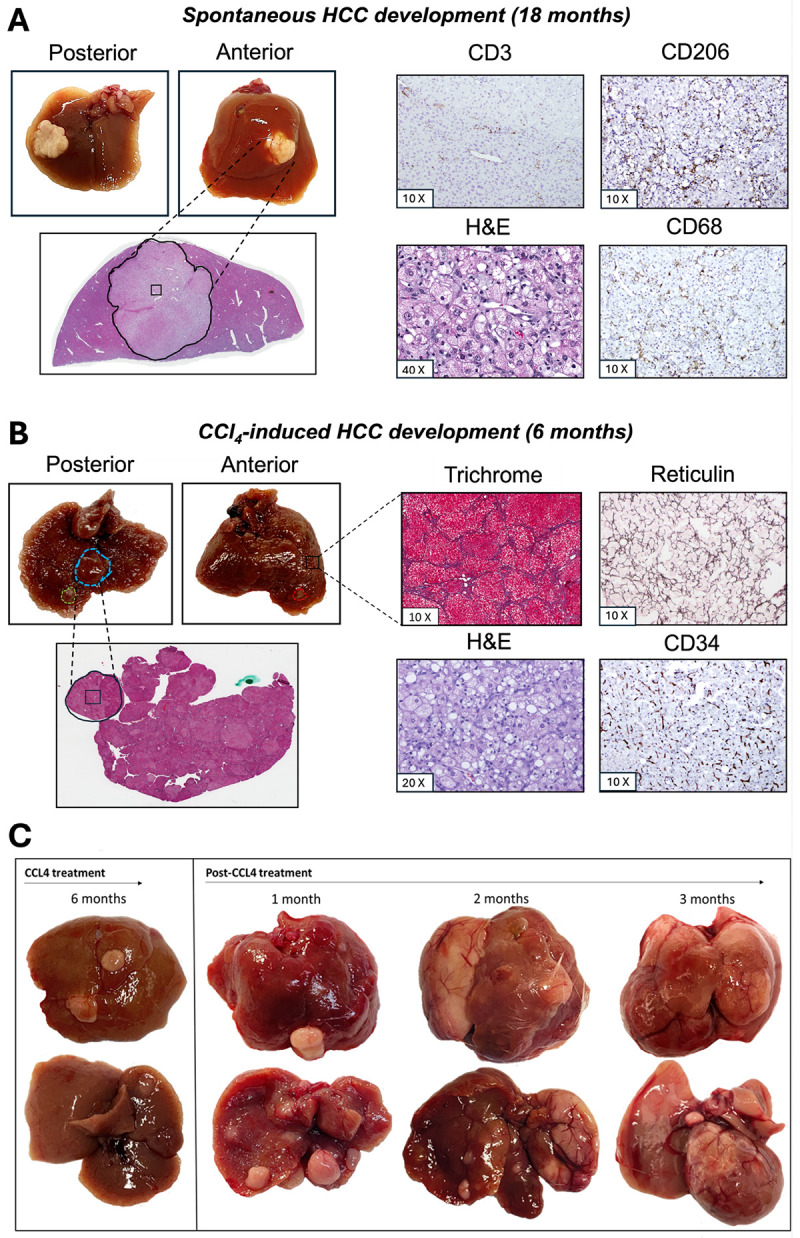
Comparative evolution and histopathological characterization of HCC in Mdr2^−^/^−^ mice with or without chronic CCl_4_ injury. (A) Gross liver morphology and representative histology a 1.5-year-old untreated Mdr2^−^/^−^ mouse demonstrating spontaneous HCC development in the absence of exogenous liver injury. H&E staining confirmed neoplastic tumor. Immunohistochemistry of the tumor microenvironment reveals sparse CD3^+^ T cell infiltration and predominance of CD206^+^ M2-like macrophages, with moderate CD68^+^ macrophage presence, indicative of an immunosuppressive milieu. The box represents a selected area for 10X and 20X magnifications. (B) Early tumor development in an Mdr2^−^/^−^ mouse after 6 months of chronic CCl_4_ administration. Gross pathology shows multifocal tumor nodules in both anterior and posterior lobes. Corresponding histopathological analyses reveal marked fibrosis (Masson’s Trichrome), disrupted reticulin framework, neovascularization (CD34), and viable tumor architecture on H&E. The boxes represent selected areas for 10X and 40X magnification. (C) Time-course of tumor evolution following CCl_4_ withdrawal. Tumors first emerge at 6 months of CCl_4_ exposure and persistently progress at 1-, 2-, and 3-months post-treatment cessation. Gross pathology shows increasing tumor burden and nodule size over time, indicating that established lesions continue to evolve despite removal of the fibrogenic insult.

Sample calculation for the experiments considered 6 mice randomly assigned per group, a potential dropout rate during fibrosis induction of 5%, and 90% tumor growth rate (6 mice × 2 groups × 1.05 × 1.10 = 14). 24 animals were initially used to establish CCl_4_-induced early onset of HCC in cirrhotic liver. Contrast-enhanced T_1_-weighted MR imaging confirmed CCl_4_-induced tumor growth at week 24. Animals were sacrificed and tumors with their adjacent tissues were histologically evaluated using Hematoxylin and Eosin, Masson’s Trichrome, reticulin, and CD34 stains (Fig. 2).

### Cryoablation protocol

Upon establishing the CCl_4_-induced HCC protocol, 18 additional mice were divided randomly into complete cryoablation (cCryo) (n = 6), incomplete cryoablation (iCryo) (n = 6), and a sham control group (n = 6). In this study, cCryo refers to full ablation of a single targeted tumor lesion, whereas iCryo refers to partial ablation of a single lesion with preserved viable tumor rims. Additional lesions in the multifocal Mdr2^−^/^−^ background were not targeted and served as internal controls. Cryoablation was performed using a clinical Visual-ICE^™^ Cryoablation System (Boston Scientific, Mass). A 1.2 mm surface cryoprobe (Galil Medical ICEx Cryoablation System, Boston Scientific) was placed on the laparoscopically exposed target lesion with careful isolation of adjacent structures. A total of two freezing cycles at the target temperature of −40°C were applied for 50 seconds of active freezing with a one minute break between cycles. The probe was placed between 80–90° degrees with its tip reaching the middle of the tumor’s long axis to ensure cCryo or iCryo. After completion of the freeze-thaw cycle, a few drops of a warm saline solution were instilled into the abdominal cavity, and the fascia, subcutaneous tissue, and skin were closed in separate layers (see Appendix E1 for additional details).

### pH_e_ imaging with BIRDS

To evaluate the metabolic impact of cryoablation on HCC tumors, we conducted pH_e_ mapping on CCl_4_-treated tumor-bearing animals both before (n = 6) and after cCryo (n = 6) or iCryo (n = 6). MRI and MR Spectroscopic Imaging (MRSI) data were acquired using a 9.4T Bruker horizontal bore spectrometer (Billerica, MA, USA) with a surface coil (12 mm diameter) tuned to ^1^H frequency (400.55 MHz). Anatomical T_1_-weighted images were obtained using a field of view (FOV) of 25mm × 25mm and 15 slices of 0.5 mm thickness. Following the anatomical MRI scans, we performed BIRDS to measure pH_e_. During the procedure, the animals were maintained on a heating pad to keep the body temperature in the 36–37°C range and were maintained under anesthesia with 1–2% isoflurane and mechanically ventilated (70% N2O; 30% O_2_). An intraperitoneal line (PE10, Braintree Scientific, LLC) was inserted for the administration of probenecid and TmDOTP^5−^. For pH_e_ mapping, the animals first received an intraperitoneal bolus injection of probenecid at 100 mg/kg, followed by a co-infusion of 140 mg/ml TmDOTP^5−^ (Macrocyclics, Dallas, TX, USA) with 100 mg/kg probenecid at a rate of 120–140 μL/h for 90 minutes. Probenecid, an organic anion transport inhibitor, was used to slow the renal clearance of contrast agents, temporarily increasing the TmDOTP^5−^ concentration in the blood. BIRDS datasets were collected with a FOV of 19mm×15mm×25mm, 256 averages, 623 spherical encoding steps, and a total acquisition time of 16 minutes.

### Data processing

**Figure 3:**
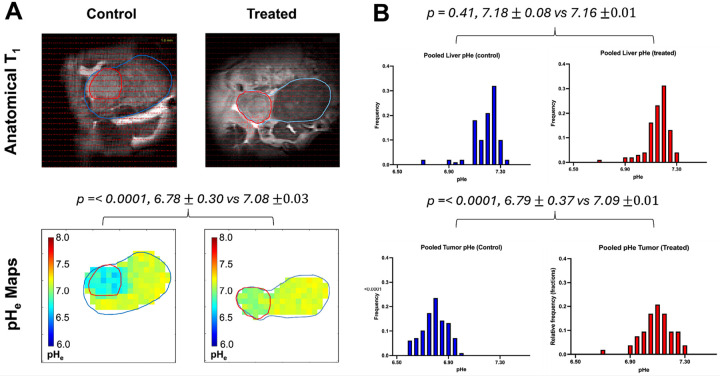
BIRDS reveals intratumoral pH_e_ re-normalization induced by cryoablation. **(A)** Representative voxel-wise pH_e_ maps overlaid on T_1_-weighted anatomical MR images from control (left) and cryoablation-treated (right) mice. Tumor regions (red contour) exhibit marked acidity in control animals, whereas treated tumors show significantly more neutral pH_e_ values. Liver parenchyma (blue contour) appears pH_e_-stable between groups. Quantitative voxel-wise analysis demonstrated significant tumor pH_e_ elevation in treated mice compared to controls (mean ± SD: 7.08 ± 0.03 vs. 6.78 ± 0.30; *p* < 0.0001), while liver pH_e_ remained comparable (7.16 ± 0.01 vs. 7.18 ± 0.08; *p* = 0.41). **(B)** Histograms showing pooled voxel-wise pH_e_ distributions across all animals for liver and tumor regions in control (blue) and cryo-treated (red) groups. Treated tumors display a right-shifted pH_e_ distribution consistent with reduced acidosis and tumor metabolic reprogramming following ablation.

**Figure 4:**
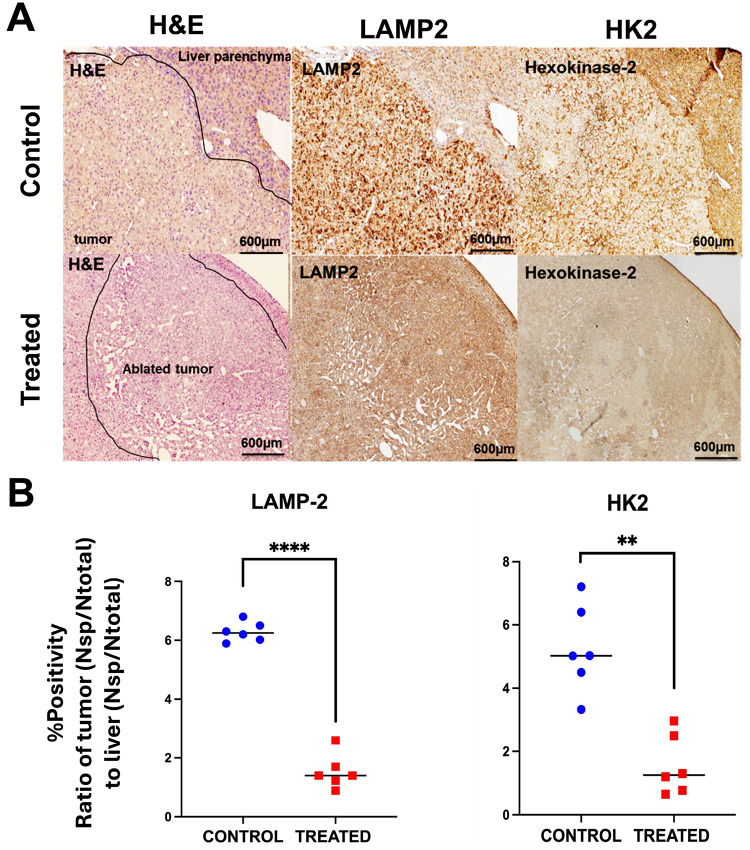
Cryoablation reduces metabolic marker expression in HCC. **(A)** Representative histological (H&E) and immunohistochemical (IHC) staining of liver and tumor tissues from untreated and cryoablation-treated Mdr2^−^/^−^ mice. In untreated animals, tumors display preserved morphology and robust staining for both LAMP-2 (a lysosomal marker) and hexokinase-2 (HK2; a glycolytic enzyme), indicative of intact tumor cell viability and metabolic activity. In contrast, cryoablated tumors show histological evidence of necrosis with markedly decreased expression of both LAMP-2 and HK2, consistent with metabolic suppression in the ablated regions. **(B)** Quantitative histopathological analysis of IHC staining expressed as the ratio of tumor-to-liver % positivity (normalized specific positivity, NSP) for LAMP-2 and HK2. Cryoablation significantly reduced both LAMP-2 (untreated vs. treated: 6.285 ± 0.21 vs. 1.53 ± 0.27; ****p < 0.0001) and HK2 expression (5.24 ± 0.68 vs. 1.56 ± 0.25; **p < 0.01), confirming downregulation of tumor-associated lysosomal and glycolytic activity post-treatment. Data are presented as mean ± SD.

**Figure 5:**
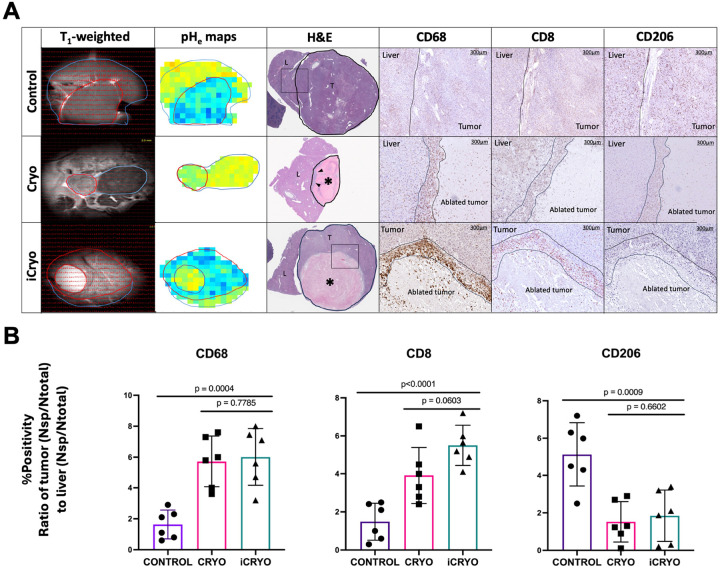
Radiological-pathological correlation demonstrates that cryoablation neutralizes inherent tumor acidosis and restores immune permissiveness. (A) Representative multi-modal MR imaging and histological assessment of HCC tumors treated with complete cryoablation (cCryo), incomplete cryoablation (iCryo), or no treatment (Control). T_1_-weighted anatomical MRI (left column) and corresponding pH_e_ maps (second column) illustrate tumor segmentation (red), liver parenchyma (blue), and changes in regional tumor acidity post-treatment. In the iCryo group (cryoablated area delineated in black), peripheral tumor zones remained acidic relative to the neutralized tumor core. H&E staining (third column) demonstrates preserved tumor morphology in untreated animals and evidence of necrosis (*) in cryoablated tumors. In cCryo, arrows indicate reminiscent tumor tissue and presence of immune infiltrate at the transitional zone between tumor and liver. IHC staining for CD68 (macrophages), CD8 (cytotoxic T cells), and CD206 (M2-like macrophages) across the three groups (right columns) reveals marked immune remodeling after cryoablation. cCryo and iCryo groups exhibited increased CD68^+^ and CD8^+^ immune cell infiltration at the tumor-liver interface, with a concomitant reduction in CD206^+^ macrophages compared to untreated group. (B) Quantification of immune cell markers in tumor tissue relative to adjacent liver, expressed as normalized specific positivity (NSP) ratio (tumor/liver) shows that both cCryo and iCryo significantly increased CD68^+^ macrophage infiltration compared to untreated group (p = 0.0004), while CD8^+^ T cell infiltration was slightly more elevated in the iCryo groups (cCryo vs. Untreated, p < 0.0001; iCryo vs. Untreated, p < 0.0001). A significant reduction in CD206^+^ macrophages was observed in both treatment conditions (cCryo vs. Untreated, p = 0.0009; iCryo vs. Untreated, p = 0.0009). Bars represent mean ± SD.

The BIRDS datasets were reconstructed to a 19×15×25 matrix, corresponding to a 1mm^3^ isotropic voxel resolution. The pH_e_ in each voxel was calculated in MATLAB (MathWorks, Inc., Natick, MA) from the ^1^H chemical shifts δ2, δ3, and δ6 of the H2, H3, and H6 protons of TmDOTP^5−^, respectively, as previously described. The BIRDS signals were overlaid on the T_1_-weighted images for anatomical localization (Fig. 3). pH_e_ was measured for voxels within the entire tumor, at the tumor edge (including voxels either adjacent to or partially outside the tumor boundary), and outside the tumor within the liver parenchyma. The region of interest (ROI) for normal liver tissue was typically selected one or two slices away from the tumor.

### Tissue harvest and processing

Tumor tissues and spleen were harvested, fixed in 10% formalin (Avantik Biogroup, Cat. No. UN334) at 4°C, paraffin-embedded, and sectioned into 5 μm-thick slices. H&E staining was performed for histopathological analysis. Immunohistochemistry (IHC) was performed using specific antibodies targeting T lymphocytes (CD3^+^ and CD8^+^), macrophages (CD68^+^), and M2-polarized macrophages (CD206^+^) to evaluate immune cell infiltratration. Metabolic markers of acidosis and glycolytic activity were evaluated using the lysosomal marker LAMP-2 and the glycolytic enzyme hexokinase-2 (HK2), respectively. Masson’s Trichrome staining was used for evaluation of cirrhosis (Fig. E1).

### Imaging analysis

Samples were digitized at 20X magnification (3.94 × 10^6^ pixels = 1 mm^2^) and percent cell positivity was assessed with ImageScope v12.3 software (Leica Biosystems Imaging, Inc., Vista, CA) using the software’s positive pixel count algorithm^[34]^. The evaluation of cirrhosis was based on the presence of architectural hepatic distortion, regenerative nodules, and fibrotic septa. A board-certified pathologist (X.X.) confirmed the stage of cirrhosis across samples. Fibrosis index was calculated as the ratio of fibrosis area to total sample area (in pixels), automatically quantified using the Leica Aperio Image Scope Positive Pixel Count (PPC) algorithm (see Appendix E1 for additional details).

### Statistical analysis

Data were analyzed using GraphPad Prism (Version 10.0, GraphPad Software, San Diego, California USA). Descriptive statistics are presented as mean ± standard deviation (SD). Unpaired two-tailed unpaired Student’s t-test with Welch’s correction was applied for comparison of continuous variables. A two-tailed *p*-value < 0.05 was considered statistically significant. Analysis of variance (ANOVA) with Bonferroni test was performed for comparison of more than 2 groups. No mice were excluded from the analyses.

## Supplementary Material

Supplementary Files

This is a list of supplementary files associated with this preprint. Click to download.

- manuscriptsupplementarymethodsNatureCommunications.docx

## Data Availability

The data that support the findings of this study are available from the corresponding author upon reasonable request. Raw MR spectroscopic data are archived at the Yale Magnetic Resonance Research Center and can be made available for academic research purposes upon reasonable request. Custom MATLAB code used for voxel-wise pHe maps and analysis is available from the corresponding author upon reasonable request.
